# The Role of Personality Traits in Nursing Students’ Attitudes Toward Artificial Intelligence

**DOI:** 10.7759/cureus.78847

**Published:** 2025-02-11

**Authors:** Areti Tsiara, Vissarion I Bakalis, Aikaterini Toska, Sofia Zyga, John D Stathoulis, Eleni N Albani, Maria Saridi, Constantinos Togas, Michail Agraniotis, Evangelos C Fradelos

**Affiliations:** 1 Primary Health Care, University of Thessaly, Larissa, GRC; 2 Nursing, University of Thessaly, Larissa, GRC; 3 Nursing, Laboratory of Clinical Nursing, University of Thessaly, Larissa, GRC; 4 Nursing, University of Peloponnese, Tripolis, GRC; 5 Biomedical Engineering, University of Peloponnese, Sparta General Hospital, Sparta, GRC; 6 Nursing, University of Patra, Patra, GRC; 7 Psychology, Panteion University, Athens, GRC

**Keywords:** artificial intelligence and education, attitudes and beliefs, bachelor’s nursing students, personality inventory, student nurses

## Abstract

Aim: This study assesses nursing students’ attitudes toward artificial intelligence (AI) and examines the role of personality traits in shaping these attitudes.

Methods: A cross-sectional study was conducted in which 159 nursing students from the University of Thessaly participated. Data were collected using the General Attitudes Toward Artificial Intelligence Scale (GAAIS) to measure attitudes toward AI and the Ten Item Personality Inventory (TIPI) to assess personality traits. Statistical analysis included descriptive and inferential methods, such as correlation and factor analysis. The significant level was set to p<0.05.

Results: The findings revealed moderately positive attitudes toward AI (mean positive attitude score: 3.22 out of 5). Extraversion and openness to experience were positively correlated with positive attitudes, while maternal education was significantly associated with lower negative attitudes.

Conclusion: Nursing students demonstrate a cautious optimism toward AI, with personality traits and education playing a key role in shaping their perceptions. Addressing concerns about AI through targeted educational programs could enhance students’ confidence and willingness to adopt AI in their professional practice. These findings emphasize the importance of integrating AI into nursing curricula to bridge knowledge gaps and promote the effective use of AI technologies in healthcare.

## Introduction

Artificial intelligence (AI) has emerged as a powerful tool in healthcare offering new opportunities and challenges. However, the acceptance and attitude of nursing students toward AI are crucial for its effective integration into the discipline. Understanding the factors that influence these attitudes, particularly the personality traits of new students, is an important area of research. According to recent studies, nursing students generally exhibit ambivalence toward AI [1,2], despite acknowledging its potential to improve healthcare delivery. They express concerns about the loss of the human dimension in care and the possibility of job loss. According to Labrague et al. (2023) [1], who analyzed the attitudes of 200 nursing students, it was found that 60% of the participants were skeptical about AI due to a lack of education and knowledge. Furthermore, a study by Lukić et al. (2023) involving 336 students showed that acceptance of AI was higher among those who had more exposure to technological tools during their studies [2].

In the field of nursing, AI has the potential to improve patient care, reduce workload, and support clinical decision-making [3]. However, the acceptance of AI depends on nurses' attitudes, which are shaped by a multitude of factors such as knowledge, experience, ethical concerns, and education. Nurses' attitudes toward AI differ, and these differences are linked to their lived experience with technology and the education they have received in their working environment [4]. For example, nurses working in environments with advanced technology are more likely to accept AI and integrate it into their daily work, whereas those in environments with limited resources show greater resistance. Nurses often express concerns about the role and impact of AI in nursing, as well as its effect on human care. Understanding their perceptions and attitudes is critical for the successful integration of these technologies [5].

Nurses who have experience using AI in clinical practice are much more positive about implementing the technology than those who are not familiar with AI tools [6]. Furthermore, the study by Georgadarellis et al. shows that 70% of nurses believe AI can enhance their efficiency [7]. However, 45% are concerned about the reduction of human contact. One of the main concerns among nurses is that the use of AI may reduce human contact and empathy in patient care. Nursing relies on building relationships of trust and emotional support, which are difficult for machines to replicate [8,9]. Additionally, nurses are often concerned about the ethical dimensions of AI, such as the security of patient data and the possibility of incorrect decisions by algorithms. In addition, questions arise about who is responsible in cases of error: the nurse or the AI system [10]. At the same time, many nurses express doubts about their ability to use complex technologies, especially when they have not received adequate training [11]. Resistance to change is common, especially in settings where AI is perceived as a threat to jobs or work routines [12].

Therefore, nurses' perceptions and attitudes toward AI are shaped by a number of factors that may influence their willingness to integrate these technologies into their daily practice. Key factors influencing their attitudes and perceptions include education, experience, and cultural factors. Education is an important factor that enhances nurses' confidence in AI. At the same time, personality traits play a significant role in students' attitudes toward AI. The relationship between personality and attitudes can help in developing educational programs that promote positive acceptance of AI. More specifically, the study by Kaya et al. (2024) analyzed the relationship between personality traits (according to the Big Five model) and acceptance of AI [13]. It was found that conscientiousness and receptivity to new experiences were significant factors that positively influenced attitudes toward AI. Additionally, according to a meta-analysis by Riedl (2022) [14], 58 empirical studies showed that people with high levels of neuroticism had more negative attitudes toward AI, while extroverted individuals were more willing to accept it [14].

Attitudes toward computers and information and communication technologies (ICT) have been extensively studied in Greece, with research providing a clear picture of users' perceptions and experiences, especially in the educational and professional fields [15-17]. However, attitudes toward AI remain less explored, particularly in the field of nursing education. The lack of appropriately weighted tools to accurately measure attitudes toward AI in Greece is a significant barrier to understanding public views and concerns. This gap limits the possibility for comparative analysis and the development of targeted educational interventions. Moreover, it is poorly understood which personality traits are related to attitudes toward AI, creating a need for further research. There is an urgent need to develop valid and reliable tools that allow for effective assessment of attitudes and perceptions, contributing to the understanding of how the public perceives and interacts with AI. Such tools could support the application of AI in areas such as healthcare, promoting its acceptance by future nursing professionals.

The purpose of this study is to explore and gain an in-depth understanding of the relationship between nursing students' perceptions and attitudes toward AI, and how their characteristics influence these perceptions and attitudes. In addition, the psychometric properties and structural validity of the General Attitudes Toward Artificial Intelligence Scale (GAAIS) will be examined.

## Materials and methods

Study design

A cross-sectional study design was employed for this study. Data collection took place at the Department of Nursing, Faculty of Health Sciences, University of Thessaly, from October 14-18, 2024, during the winter semester of 2024-2025. The sample data were collected using an anonymous, fully structured, self-completed questionnaire. The questionnaires were distributed in hard copy by the author in classrooms within the university, with verbal instructions given to students about the purpose of the study and how to complete the questionnaire. Additionally, the researcher provided clarification for any questions to ensure participants received clear and complete information. This approach enhanced the accuracy of the protocol. The completed questionnaires were personally returned to the researcher in a sealed envelope to ensure data confidentiality. The time required to complete the questionnaire did not exceed 15 minutes, ensuring no significant burden on the participants regarding their academic duties. Participation in the survey was voluntary, following an explanation of the study's objectives and assurance of anonymity. Verbal consent was obtained to ensure ethical compliance, and participants were informed that they could withdraw consent at any time without needing to provide reasons for their decision. A total of 170 questionnaires were administered, and 159 completed questionnaires were returned, yielding a 93% response rate.

Data collection tools

The measurement tool used consisted of three parts.

The Nursing Students’ Individual Characteristics Recording Sheet

It included questions about demographics, as well as social and academic characteristics, such as gender, age, year of study, outstanding courses, origin, marital status, parents' educational level, family financial status, work alongside studies, sexual orientation, and use of social media.

General Attitudes Toward Artificial Intelligence Scale

The GAAIS consists of two subscales that assess different aspects of attitudes toward AI, such as perceptions of the benefits of AI, concerns and fears about AI, and general acceptance and willingness to use AI. These may include computers, robots, or other hardware devices possibly augmented with sensors or cameras, etc. The response options are: Strongly Disagree, Disagree, Partially Disagree, Neutral, Partially Agree, and Totally Agree. Participants may also choose "I prefer not to answer" for ethical reasons. Each participant selects one response per question.

The scale has two sub-dimensions: a. Positive attitude toward AI (items: 1, 2, 4, 5, 7, 11, 12, 13, 14, 16, 17, 18). These items are scored from 1 (Strongly Disagree) to 5 (Strongly Agree), with Neutral at 3. This sub-dimension reflects enthusiasm, optimism, and acceptance of AI, focusing on benefits, societal contributions, and its potential to simplify life. b. Negative attitude toward AI (items: 3, 6, 8, 9, 10, 15, 19, 20). These items are reverse-scored (1 = Strongly Agree, 5 = Strongly Disagree). They assess concerns about AI, including job loss, moral issues, and over-reliance on technology. For analysis, the scale is inverted, meaning higher values indicate lower concern or negativity. Thus, a higher score on each subscale represents a more positive attitude toward AI [18,19].

Assessing Personality Traits Using the Ten Item Personality Inventory Tool

Personality traits were assessed using the Ten Item Personality Inventory (TIPI), a personality assessment tool designed to measure the five major personality dimensions, known as the Big Five (Gosling et al., 2003). These dimensions are extraversion (items 1 and 6), agreeableness (items 2 and 7), conscientiousness (items 3 and 8), emotional stability (items 4 and 9), and openness to experiences (items 5 and 10). The TIPI consists of 10 questions, with two questions assessing each of the five dimensions. Each question uses a seven-point Likert scale, ranging from "Strongly Agree" to "Strongly Disagree" [20].

Statistical analysis

The processing and statistical analysis of the empirical data for the research were conducted using the software package SPSS (Statistical Package for the Social Sciences) 29.0 for Windows (IBM Corp., Armonk, New York, USA) with descriptive and inferential statistical methods. Specifically, the descriptive analysis included the frequency distribution of the variables (absolute and relative percentage frequency) and estimates of location and dispersion parameters for the quantitative variables (mean, standard deviation, median, maximum, and minimum values). Inferential analysis to investigate potential correlations included Pearson's correlation coefficient (r), Spearman's Rho (ρ), Chi-Square test, t-test, one-way ANOVA for independent samples, and Bonferroni's test for multiple comparisons. Additionally, factor analysis was performed on the GAAIS. The significance levels were two-sided, with an acceptable level of statistical significance set at p<0.05.

Ethics

The research ethics and ethics framework were strictly followed in the organization and conduct of the research, as outlined by the Research Ethics Committee of the University of Thessaly. This committee operates in accordance with the provisions of Law 4957/2022, Articles 277-282, and approval was obtained for the thesis topic and the conduct of this research. Specifically, the required rights and permissions for the use of the questionnaires were obtained from the supervising professor: i) GAAIS, and ii) TIPI. This study was approved by the Nursing Department Research Ethics Committee (approval No. 753/02-09-2024).

## Results

Table 1 presents the demographic characteristics of the sample, consisting of 159 participants. The majority of participants were female (70.4%), with a mean age of 20.9 years (SD = 3.5) and an average year of study of 3.1 (SD = 0.9). Most students had failed between 0 and 2 courses (78%). Regarding marital status, more than half were single (53.5%), and 42.1% were in a relationship. Over half of the participants resided in urban areas (55.3%), with the majority living alone (56%). The educational background of fathers and mothers showed higher representation at the university level (50% and 57.6%, respectively). Household income predominantly fell between €10,000 and €25,000 per year (57.9%). Over half of the sample (53.5%) reported being employed. The majority identified as heterosexual (89.9%), with a small percentage identifying as homosexual, bisexual, or other. Social media use varied, with half of participants spending 2-5 hours per day (50.9%) and 27% exceeding 5 hours daily.

**Table 1:** Demographic characteristics of the sample, n=159

Variable	Category	Number (n)	Percentage (%)
Gender	Male	46	28.9
	Female	112	70.4
	Other	1	0.6
Failed courses	0-2	124	78
	3-6	24	15.1
	>7	11	6.9
Marital status	Single	85	53.5
	In relationship	67	42.1
	Married	7	4.4
Residence	Rural	41	25.8
	Semi-urban	30	18.9
	Urban	88	55.3
Living arrangement	Alone	89	56
	With parents	38	23.9
	Cohabited	32	20.1
Father's education	Elementary	12	7.6
	High school	67	42.4
	University	79	50
Mother's education	Elementary	9	5.7
	Highschool	58	36.7
	University	91	57.6
Income	<10.000 euro/year	30	18.9
	10.000-25.000 euro/year	92	57.9
	>25.000 euro/year	37	23.3
Working	Yes	85	53.5
	No	74	46.5
Sexual orientation	Heterosexual	143	89.9
	Homosexual	5	3.1
	Bisexual	7	4.4
	Other	4	2.5
Time spent in social media	>5 h/day	43	27
	2-5 h/day	81	50.9
	<2 h/day	30	18.9
	Rarely	5	3.1

Table 2 presents the descriptive statistics for the scales used in the study. The mean score for positive attitudes toward AI was 3.224 (SD = 0.515), with a range from 1.917 to 4.583, and good internal consistency (Cronbach’s α = 0.790). Negative attitudes toward AI had a mean score of 2.823 (SD = 0.757), ranging from 1.375 to 7.875, with moderate reliability (Cronbach’s α = 0.670). Among the personality traits, extraversion scored a mean of 8.063 (SD = 2.855) with Cronbach’s α = 0.571, while agreeableness had a mean of 10.371 (SD = 2.130), but showed low reliability (Cronbach’s α = 0.243). Conscientiousness had a mean of 10.516 (SD = 2.441) and moderate reliability (Cronbach’s α = 0.577). Emotional stability, with a mean of 8.302 (SD = 5.419), demonstrated poor reliability (Cronbach’s α = 0.016), as did openness to experiences, which had a mean of 10.582 (SD = 2.201) and Cronbach’s α = 0.319. These findings indicate differences in internal consistency across the scales. In particular, emotional stability and agreeableness showed lower reliability.

**Table 2:** Mean score, SD, min-max, and reliability for each study scale

Personality traits	Mean	SD	Minimum	Maximum	Cronbach a
Positive attitudes toward AI	3.224	0.515	1.917	4.583	0.790
Negative attitudes toward AI	2.823	0.757	1.375	7.875	0.670
Extraversion	8.063	2.855	2.000	14.000	0.571
Agreeableness	10.371	2.130	3.000	14.000	0.243
Conscientiousness	10.516	2.441	4.000	14.000	0.577
Emotional stability	8.302	5.419	2.000	69.000	0.016
Openness to experiences	10.582	2.201	2.000	14.000	0.319

Table 3 presents the results of the confirmatory factor analysis for the GAAIS. The model demonstrated an acceptable Chi-square/df ratio of 1.777, indicating a good fit. Although the model's p-value was significant (p = 0.001), which may suggest a lack of perfect fit, other indices provide further insights. The comparative fit index (CFI) was 0.80, reflecting a moderate fit. The goodness of fit index (GFI) was high at 0.984, suggesting that the model closely matches the observed data. The standardized root mean square residual (SRMR) was 0.083, within acceptable limits, and the root mean square error of approximation (RMSEA) was 0.07, also indicating a reasonable fit. Overall, the analysis supports the two-factor model, which has an adequate fit but leaves room for improvement in some indices, particularly the CFI.

**Table 3:** Goodness of fit indicators for the GAAIS model GAAIS, General Attitudes Toward Artificial Intelligence Scale

Indicator	Value
Chi-square/df	1.777
Model p-value	0.001
Comparative fit index	0.800
The goodness of fit index	0.984
Standardized root means square residual	0.083
Root mean square error of approximation	0.070

Table 4 explores the relationship between demographic characteristics and attitudes toward AI. Gender differences in positive attitudes showed no significant differences (t = 1.637, p = 0.104), while age and year of study were weakly correlated with positive attitudes (r = 0.127, p = 0.122; r = 0.152, p = 0.064, respectively). Analysis of failed courses, marital status, residence, living arrangements, and parents' education revealed no significant differences in positive attitudes toward AI. However, maternal education was significantly associated with negative attitudes (F = 4.771, p = 0.010), with elementary education linked to more negative attitudes compared to university-level education (Bonferroni-adjusted p = 0.015). Income, employment status, and time spent on social media did not significantly influence attitudes. These findings suggest that demographic factors may have a limited impact on attitudes toward AI, while educational background, particularly maternal education, might play a more influential role in shaping perceptions.

**Table 4:** Relationship between demographic characteristics and attitude toward AI AI, artificial intelligence

		Positive attitude, mean (SD)	Test statistic	p-value	Negative attitude, mean (SD)	Test statistic	p-value
Gender							
Male		3.33 (0.51)	1.637	0.104	2.84 (0.65)	t = 0.204	0.839
Female		3.18 (0.51)			2.81 (0.79)		
Age		r = 0.127		0.122	r = 0.016		0.850
Year of study		r = 0.152		0.064	r = 0.015		0.859
Failed courses							
0-2		3.23 (0.51)	F = 0.904		2.76 (0.63)	F = 1.816	0.166
3-6		3.10 (0.51)			3.11 (1.28)		
>7		3.33 (0.58)			2.92 (0.62)		
Marital status							
Single		3.23 (0.52)	F = 1.816	0.166	2.82 (0.86)	F = 0.037	0.964
In relationship		3.21 (0.50)			2.81 (0.63)		
Married		3.14 (0.52)			2.89 (0.52)		
Residence							
Rural		3.12 (0.49)	F = 1.601	0.205	2.72 (0.72)	F = 0.505	0.605
Semi-urban		3.35 (0.54)			2.87 (0.59)		
Urban		3.22 (0.51)			2.77 (1.06)		
Living arrangement							
Alone		3.29 (0.50)	F = 2.183.	0.116	2.71 (0.57)	F = 2.200	0.115
With parents		3.17 (0.52)			3.00 (1.02)		
Cohabited		3.07 (0.49)			2.92 (0.67)		
Father's education							
Elementary		3.23 (0.60)	F = 0.560	0.205	3.29 (1.60)	F = 2.527	0.084
High school		3.17 (0.55)			2.74 (0.63)		
University		3.26 (0.47)			2.81 (0.63)		
Mother's education							
Elementary (I)		3.19 (0.43)	F = 0.538	0.585	3.54 (1.67)	F = 4.771	0.010
Highschool (II)		3.16 (0.56)			2.71 (0.63)		I>III, 0.015
University (III)		3.26 (0.49)			2.80 (0.64)		
Income							
<10.000 euro/year		3.12 (0.55)	F = 0.721	0.488	2.98 (1.10)	F = 0.883	0.416
10.000-25.000 euro/year		3.26 (0.48)			2.75 (0.75)		
>25.000 euro/year		3.19 (0.55)			2.79 (0.59)		
Working							
Yes		3.21 (0.55)	t = -0.177	0.859	2.92 (0.57)	t = -1.645	0.102
No		3.23 (0.47)			2.71 (0.89)		
Time spent in social media							
>5 h/day		3.23 (0.68)	F = 1.012	0.389	2.75 (0.79)	F = 1.907	0.131
2-5 h/day		3.32 (0.52)			2.64 (0.57)		
<2 h/day		3.17 (0.40)			2.57 (0.80)		
Rarely		3.01 (0.65)			5.57 (0.87)		
t = Student's t-test, F = ANOVA, r = Pearson correlation coefficient							

Pearson's correlation analysis (Table 5) reveals relationships between attitudes toward AI and personality traits. A significant negative correlation was observed between positive and negative attitudes toward AI (r = -0.233, p = 0.006), indicating that individuals with more positive attitudes tend to have fewer negative attitudes. Extraversion was positively, though not significantly, correlated with positive attitudes (r = 0.075, p = 0.363) and negatively correlated with negative attitudes (r = -0.181, p = 0.030). Agreeableness, conscientiousness, and emotional stability showed weak and non-significant correlations with both positive and negative attitudes. Openness to experience was positively correlated with positive attitudes (r = 0.166, p = 0.043) but negatively and non-significantly correlated with negative attitudes (r = -0.137, p = 0.102). These findings suggest that personality traits, particularly extraversion and openness to experience, may influence attitudes toward AI, albeit modestly.

**Table 5:** Pearson's correlations between attitudes toward AI and personality traits AI, artificial intelligence

Variable		Positive attitudes toward AI	Negative attitudes toward AI
Negative attitude toward AI	Pearson's r	-0.233	-
	p-value	0.006	-
Extraversion	Pearson's r	0.075	-0.181
	p-value	0.363	0.030
Agreeableness	Pearson's r	-0.083	-0.039
	p-value	0.312	0.640
Conscientiousness	Pearson's r	-0.030	-0.157
	p-value	0.713	0.061
Emotional Stability	Pearson's r	0.130	-0.086
	p-value	0.113	0.307
Openness to Experiences	Pearson's r	0.166	-0.137
	p-value	0.043	0.102

## Discussion

This study highlighted nursing students' attitudes toward AI, addressing both positive and negative aspects. The results indicate that students generally held moderately positive attitudes toward AI. Higher positive attitudes were associated with lower negative attitudes, suggesting that as students viewed AI more favorably, their concerns about it decreased. In terms of personality traits, Extraversion and openness to experience were positively associated with positive attitudes, although the correlations were moderate. In contrast, the other personality traits did not show significant correlations.

Understanding nursing students' attitudes toward AI is critical for integrating these technologies into clinical practice. Studies have shown that nursing students have positive perceptions of the use of AI in nursing practice and express a high intention to adopt this technology [1]. Furthermore, integrating AI into nursing education can provide personalized and effective learning experiences, preparing students with the skills needed in the modern workplace [21]. However, there are concerns about the loss of the human dimension of care and the lack of appropriate training. Nursing students may benefit from educational programs that focus on the applications of AI in nursing, enhancing their knowledge and confidence in this technology [2]. Promoting positive attitudes toward AI can help create a more efficient and technologically aware nursing workforce, ready to take full advantage of the opportunities offered by AI in healthcare [22].

Regarding the structural construction of the scale, the analysis supports the bivariate model with a sufficient fit, although some CFI indicators suggest room for improvement. Comparing these results with those of other studies, we note that they align with the original study on the development and validation of the Attitudes Toward Artificial Intelligence Scale (AIAS-4). The authors reported that the scale was designed to capture attitudes toward AI, focusing on the perceived usefulness and potential impact of the technology on society and humanity [18,19]. The structural construction of the scale has been validated in other languages [23]; confirmation of the original structure suggests that the questionnaire is robust and sufficiently designed to capture the public's attitudes toward AI in different contexts.

The survey results suggest that participants hold slightly more positive attitudes toward AI, with positive perceptions outweighing negative concerns. However, the greater variance in negative attitudes indicates that views on the risks or challenges of AI vary considerably. According to Schepman and Rodway's (2020) study, the bivariate structure of the GAAIS is effective in capturing the ambivalence that often accompanies acceptance of AI, which is critical for understanding reservations. Research by Longoni et al. (2022) highlights that acceptance of AI is influenced by the extent to which users perceive it as complementary, rather than competing, with human skills [24].

At the same time, the lack of sufficient knowledge or experience with AI, as reported by the participants, can reinforce negative attitudes, confirming the need for educational interventions. Therefore, the findings of the present study emphasize the importance of targeted education and awareness-raising to manage perceptions of AI, focusing on support rather than replacement. This clarification, that AI is meant to support and enhance the work of nurses, not replace them, may help alleviate some of the concerns [25].

The analysis suggests that extroverted individuals may be less prone to negative perceptions of AI, while conscientious individuals might also hold fewer negative views, though this relationship is not significant. Additionally, those with higher openness to experience are more likely to have positive attitudes toward AI. According to Schepman and Rodway's (2022) study, openness to new experiences is associated with more positive attitudes toward AI, confirming the present findings [19]. Furthermore, Calluso and Devetag's (2024) research highlights that extroversion and openness are associated with greater acceptance and willingness to use new technologies, including AI [26]. Finally, the study by Stein et al. (2024) reports that conscientiousness may act as a protective factor against negative attitudes toward AI, supporting the negative association observed in the present analysis [27]. Overall, the findings suggest that personality traits such as extraversion, openness to experience, and conscientiousness play a role in shaping attitudes toward AI. Understanding these relationships may contribute to the development of strategies that promote more positive perceptions and greater acceptance of AI in different populations.

The integration of educational programs on AI in nursing can improve patient management and decision-making, enhancing nurses' clinical skills. The development of such programs will help increase efficiency and accuracy in care delivery [28]. In addition, exploring the role of personality in shaping nurses' professional attitudes is crucial. Personality traits, such as extraversion or emotional intelligence, influence how nurses interact with patients and respond to situations of intense emotional or psychological stress. Thus, training that combines the use of AI with an understanding of personality can lead to more integrated and person-centered care.

Among the strengths of this study is that it explores the relationship between personality traits and attitudes toward AI, offering valuable insights into how individual differences shape perceptions of technology. In addition, it examines both positive and negative attitudes, allowing for a balanced understanding of how people view AI.

This study has some limitations that affect the results and the generalizability of the conclusions. First, the sample was limited to nursing students from a single university, which restricts the ability to generalize the findings to larger populations or different educational settings. Second, the questionnaire used to assess personality traits may have weaknesses in the reliability of some of its subscales, which could affect the validity of the results. These limitations should be considered when interpreting the findings and suggest the use of larger, more representative samples as well as more reliable measurement tools in future studies.

## Conclusions

This study examined students' attitudes toward AI and their association with demographic and personal characteristics. The main findings indicate that attitudes toward AI are positive, with females showing slightly lower positive attitudes than males, although the difference was not statistically significant. Maternal education significantly influences negative attitudes, with individuals from families with at least a university-level education showing fewer negative attitudes. The low reliability of some personality scales, such as emotional stability and agreeableness, is concerning, indicating the need to improve the measurement tools.

The study highlights the importance of personal characteristics, such as extraversion and openness to experience, as possible factors influencing attitudes toward AI, although the correlations were weak. Ultimately, the findings suggest that the influence of demographic parameters is limited and that attitudes toward AI are more strongly associated with parental education and personality. Further research is needed to deepen our understanding of the factors influencing these attitudes and to develop more reliable measurement tools, particularly for scales related to personal characteristics such as emotional stability and agreeableness.

## References

[1] Labrague LJ, Aguilar-Rosales R, Yboa BC, Sabio JB, de Los Santos JA (2023). Student nurses' attitudes, perceived utilization, and intention to adopt artificial intelligence (AI) technology in nursing practice: a cross-sectional study. Nurse Educ Pract.

[2] Lukić A, Kudelić N, Antičević V, Lazić-Mosler E, Glunčić V, Hren D, Lukić IK (2023). First-year nursing students' attitudes towards artificial intelligence: cross-sectional multi-center study. Nurse Educ Pract.

[3] Bajwa J, Munir U, Nori A, Williams B (2021). Artificial intelligence in healthcare: transforming the practice of medicine. Future Healthc J.

[4] Lora L, Foran P (2024). Nurses' perceptions of artificial intelligence (AI) integration into practice: an integrative review. J Perioper Nurs.

[5] Tuncer GZ, Tuncer M (2024). Investigation of nurses' general attitudes toward artificial intelligence and their perceptions of ChatGPT usage and influencing factors. Digit Health.

[6] Seibert K, Domhoff D, Bruch D, Schulte-Althoff M, Fürstenau D, Biessmann F, Wolf-Ostermann K (2021). Application scenarios for artificial intelligence in nursing care: rapid review. J Med Internet Res.

[7] Georgadarellis GL, Cobb T, Vital CJ, Sup FC 4th (2024). Nursing perceptions of robotic technology in healthcare: a pretest-posttest survey analysis using an educational video. IISE Trans Occup Ergon Hum Factors.

[8] Mohanasundari SK, Kalpana M, Madhusudhan U (2023). Can artificial intelligence replace the unique nursing role?. Cureus.

[9] Hassan EA, El-Ashry AM (2024). Leading with AI in critical care nursing: challenges, opportunities, and the human factor. BMC Nurs.

[10] Farhud DD, Zokaei S (2021). Ethical issues of artificial intelligence in medicine and healthcare. Iran J Public Health.

[11] Gerke S, Babic B, Evgeniou T, Cohen IG (2020). The need for a system view to regulate artificial intelligence/machine learning-based software as medical device. NPJ Digit Med.

[12] Ramadan OM, Alruwaili MM, Alruwaili AN, Elsehrawy MG, Alanazi S (2024). Facilitators and barriers to AI adoption in nursing practice: a qualitative study of registered nurses' perspectives. BMC Nurs.

[13] Kaya D (2024). The adventure of artificial intelligence in educational research from the past to the present. Sakarya Uni J Edu.

[14] Riedl R (2022). Is trust in artificial intelligence systems related to user personality? Review of empirical evidence and future research directions. Electronic Markets.

[15] Papathanasiou IV, Mantzaris D, Fradelos EC (2023). Nursing students’ computer anxiety and attitudes before and during the COVID-19 pandemic. Adv Exp Med Biol.

[16] Vozikis A, Ypofanti M, Papadopoulos I (2010). Attitudes towards the use of information and communication technologies (ICT) at work: findings from the health care sector in Greece. SPOUDAI J Econ Bus.

[17] Martzoukou K, Luders ES, Mair J, Kostagiolas P, Johnson N, Work F, Fulton C (2024). A cross-sectional study of discipline-based self-perceived digital literacy competencies of nursing students. J Adv Nurs.

[18] Schepman A, Rodway P (2020). Initial validation of the general attitudes towards Artificial Intelligence Scale. Comput Hum Behav Rep.

[19] Schepman A, Rodway P (2022). The general attitudes towards artificial intelligence Scale (GAAIS): confirmatory validation and associations with personality, corporate distrust, and General Trust. Int J Hum Comput Int.

[20] Gosling SD, Rentfrow PJ, Swann Jr WB (2003). A very brief measure of the Big-Five personality domains. J Res Pers.

[21] Glauberman G, Ito-Fujita A, Katz S, Callahan J (2023). Artificial intelligence in nursing education: opportunities and challenges. Hawaii J Health Soc Welf.

[22] De Gagne JC (2023). The state of artificial intelligence in nursing education: past, present, and future directions. Int J Environ Res Public Health.

[23] Kaya F, Aydin F, Schepman A, Rodway P, Yetişensoy O, Demir Kaya M (2022). The roles of personality traits, AI anxiety, and demographic factors in attitudes toward artificial intelligence. Int J Hum Comput Int.

[24] Longoni C, Cian L (2022). Artificial intelligence in utilitarian vs. hedonic contexts: the “word-of-machine” effect. J Mark.

[25] Şahin MG, Yıldırım Y (2024). The general attitudes towards artificial intelligence (GAAIS): a meta-analytic reliability generalization study. Int J Assess Tools Edu.

[26] Calluso C, Devetag MG (2024). The impact of technology acceptance and personality traits on the willingness to use AI-assisted hiring practices. Int J Organ Anal.

[27] Stein JP, Messingschlager T, Gnambs T, Hutmacher F, Appel M (2024). Attitudes towards AI: measurement and associations with personality. Sci Rep.

[28] Al Kuwaiti A, Nazer K, Al-Reedy A (2023). A review of the role of artificial intelligence in healthcare. J Pers Med.

